# Maternal age at maturation underpins contrasting behavior in offspring

**DOI:** 10.1093/beheco/arw073

**Published:** 2016-05-10

**Authors:** Tim Burton, Grethe Robertsen, David C. Stewart, Simon McKelvey, John D. Armstrong, Neil B. Metcalfe

**Affiliations:** ^a^Institute of Biodiversity, Animal Health and Comparative Medicine, MVLS, University of Glasgow, University Avenue, Glasgow G12 8QQ, ScotlandUK; ^b^Norwegian Institute for Nature Research, Høgskoleringen 9, NO-7034 Trondheim, Norway; ^c^Marine Scotland Science, Freshwater Fisheries Laboratory, Faskally, Pitlochry, Perthshire PH16 5LB, Scotland, UK; ^d^Cromarty Firth Fisheries Board, CKD Galbraith, Reay House, 17 Old Edinburgh Rd, Inverness IV2 3HFUK

**Keywords:** aggression, competitive ability, maturation age, metabolic rate, parental effects.

## Abstract

In species where parental care occurs primarily via the provisioning of eggs, older females tend to produce larger offspring that have better fitness prospects. Remarkably however, a relationship between age of mother and fitness of offspring has also been reported independently of effects on offspring size suggesting that there may be other factors at play. Here, using experimental matings between wild Atlantic salmon that differed in their age at sexual maturation, we demonstrate distinct size-independent variation in the behavior of their offspring that was related to the maturation age of the mother (but not the father). We found that when juvenile salmon were competing for feeding territories, offspring of early-maturing mothers were more aggressive than those of late-maturing mothers, but were out-competed for food by them. This is the first demonstration of a link between natural variation in parental age at maturation and variation in offspring behavior.

## Lay Summary

In nature, vast differences in growth or size are frequently observed among young born to mothers of different age. However, it is unknown if there can be other, more subtle differences among offspring born to young versus old mothers? In Atlantic salmon, we reveal that despite being similar in size, juveniles from younger-maturing mothers are more aggressive, but poorer at competing for food than juveniles from older-maturing mothers

## INTRODUCTION

In many organisms, an individual’s age at maturation is fundamentally linked to fitness (Roff 1992). By delaying maturation, for example, females may be able to increase their body size and thus produce more and/or larger offspring. Indeed, across a range of taxa, there is clear evidence that older, larger mothers produce larger offspring and that larger offspring tend to have higher fitness than smaller offspring (Marshall et al. 2010). However, striking relationships between maternal age and offspring development and fitness have been recorded, even after controlling for offspring size (Paitz et al. 2007; Benton et al. 2008). Moreover, there has been some suggestion that paternal age can also influence offspring traits, possibly through epigenetic modifications (Curley et al. 2011). This raises the possibility that fitness-related traits in offspring, other than their size and growth, might co-vary with parental age. Using Atlantic salmon, *Salmo salar*, a species that demonstrates substantial within-population variation in maturation age independent of size at reproduction, we tested for relationships between parental age at maturation and the physiology and behavior displayed by their offspring.

Atlantic salmon spawn in rivers and streams, where juveniles live until they “smolt” (the physiological and morphological preparation for marine life). Upon smolting, the fish migrate to sea, where most of their growth occurs, before they sexually mature and return to spawn in freshwater (Klemetsen et al. 2003). A proportion of male fish become sexually mature without ever going to sea and take part in spawning as “sneakers” (Fleming 1996), but in this study we are only considering life history variation in those fish which undertake the anadromous migration. While maturation age in salmon is partly determined by a genetic component (Gjerde 1984; Gjerde et al. 1994; Johnston et al. 2014), it is also influenced by environmental factors affecting growth in both the freshwater and marine phases of life. Smolting (and consequent seaward migration) has a direct effect on maturation age because the smolt migration occurs only during spring; fish that fail to smolt in a given spring remain in freshwater for at least another year, thereby delaying the earliest age of maturation. Given that smolting is largely regulated by a minimum body size threshold (Metcalfe 1998), faster growing juveniles are more likely to smolt and thus gain the chance to mature sexually at sea earlier than slower growing juveniles. Likewise, post-smolt marine growth is also inversely correlated with time until sexual maturation (Friedland and Haas 1996; Salminen 1997; Otero et al. 2012) and probably reflects a size- and/or physiological- specific threshold that must be reached for the maturation process to occur (Salminen 1997).

It has been reported that female salmon that mature under natural conditions at a lower age can produce offspring that are able to out-grow the progeny of later-maturing mothers, despite the tendency of their offspring to hatch at the same time of year but from smaller eggs (Burton et al. 2013). This contradicts the general expectation that, in natural populations, offspring from larger eggs should have a competitive (and hence growth) advantage over those hatching at the same time from smaller eggs (Hutchings 1991; Einum and Fleming 1999). We hypothesize that variation in both offspring competitive behaviors and energy metabolism might underpin this apparent paradox between offspring size and growth performance. Growth is determined partly by both access to food and the digestive and assimilatory abilities to convert ingested food into new tissue. It has been shown that juvenile salmon with higher metabolic rates tend to become more dominant and are thus likely to gain profitable feeding territories (Burton et al. 2011); when combined with the fact that they may also digest and assimilate meals faster this can result in higher growth rates (Burton et al. 2011). Thus, we predict that offspring originating from mothers that matured early would be more competitive and have higher rates of mass-specific energy metabolism than offspring from mothers that matured at a later age. The predicted effect of paternal age on offspring traits is unclear, but was also examined.

## METHODS

### Selection and spawning of parental stock

The offspring used in this experiment were derived from wild Atlantic salmon that had all spent the same time at sea and were of similar sizes, but differed in their age at maturation (due to differences in the time it took them as juveniles to reach the size-dependent threshold that triggers seaward migration [Metcalfe and Thorpe 1990]). Those parents that had matured earlier (=Early Maturing or EM) had migrated to sea as 2-year-old smolts, whereas late maturing parents had spent another full year in the freshwater nursery environment (=Late Maturing or LM). The protocol for the selection and spawning of parental fish followed that of Burton et al. (2013), but with some minor modifications. Wild anadromous Atlantic salmon undertaking their spawning migration were captured at the Loch na Croic fish trap on the River Blackwater, Ross-Shire, northern Scotland. At the trap site, males and females were held separately in 10 completely dark circular tanks (4 m diameter, 1.5 m deep), supplied directly with water from the River Blackwater, until they reached spawning condition. Within 16 days of their capture, we randomly selected 73 females that were ready to spawn (between the 26th and 28th November 2012), determined by netting and gently squeezing the sides of each fish to identify the presence of loose eggs within the body cavity. This group of females were judged to have spent a single winter at sea before returning to freshwater to reproduce (i.e., were one-sea-winter or 1SW fish), based on visual assessment of their body size. The fork length (*L*_*F*_, to 0.5cm) and body mass (to 0.1g) of each female was recorded prior to the stripping of their eggs, which were drained of ovarian fluid and then weighed (to 0.1g; referred to hereafter as “clutch mass”).

A subsample of eggs from each female was preserved in a 5% buffered formalin solution and later weighed (to 0.0001g, *n* = 20 per clutch) to provide an initial estimate of offspring size. The remaining eggs from each female were fertilized in vitro with sperm from 1 of 73 wild anadromous males (also judged to be 1SW on the basis of length) to create 73 full sibling families. Readings of scale samples taken from each female and male subsequently confirmed that all the spawners were 1SW fish and so were breeding for the first time after entering the marine environment. Examination of juvenile growth patterns (i.e., in freshwater) based on the scale samples revealed that 21 females and 29 males migrated to sea as 2-year-old smolts (EM fish) while 52 females and 44 males migrated as 3-year-old smolts (LM fish). We note here that the LM mothers used in the current study were 1 year older than the EM mothers. Thus, even if the EM and LM mothers originated from the same natal habitat, they potentially experienced some variation in developmental conditions (e.g., due to climatic variation from 1 year to the next). Once fertilized, the eggs were transferred to the Scottish and Southern Energy (SSE) hatchery at Contin, where they were reared as separate family groups under ambient water temperatures until the eyed stage (i.e., the stage at which developing eyes become visible in the embryo, after which the embryos are robust enough to be moved). Previously we have shown that maternal size, body condition, and relative investment in eggs can affect offspring performance in natural conditions in this study system and thus could feasibly contribute to variation in offspring behaviors (Burton et al. 2013). To minimize variation in maternal size, body condition and investment in reproduction among the 2 classes of mothers used in the experiment, we selected 20 (10 from EM mothersEM_M_, 10 from LM mothersLM_M_) of the 73 families so as to ensure these 3 maternal traits did not differ between the maturation age groups (*t*-tests comparing selected EM and LM mothers for all 3 comparisons, *P* > 0.7, see Table 1); body condition was calculated as residuals from a linear regression between somatic mass (i.e., total body mass minus clutch mass) and fork length (both variables log-transformed) for all 73 female fish; reproductive investment was similarly calculated as residuals from a regression of clutch mass against fork length (both variables log-transformed).

**Table 1:** Summary of *t*-tests comparing body size, body condition, and investment in reproduction among adult female (EM, *n* = 10; LM, *n* = 10) and male (EM, *n* = 9; LM, *n* = 11) salmon used to provide offspring for the experiment

Measurement	EM females	LM females	*t*-value	*P*-value
Body size (mm)	569.5±6.8	568.5±7.2	0.10	0.92
Body condition	−0.005±0.005	−0.004±0.007	−0.08	0.94
Reproductive investment	0.02±0.02	0.03±0.02	−0.28	0.79
	EM males	LM males		
Body size (mm)	578.3±12.0	584.6±5.9	−0.46	0.65
Body condition	−0.004±0.01	−0.003±0.01	−0.02	0.98

Mean values (±SE) for each trait are displayed for EM and LM groups. Body condition and reproductive investment were calculated as residuals from regressions of somatic mass and clutch mass on body size respectively (all variables log-transformed) from a larger sample of 73 individuals. Investment in reproduction was not measured for male fish.

Although our focus was primarily on the relationship between maternal maturation age and offspring phenotype, the families chosen for the experiment were also evenly represented with respect to patterns of paternal maturation age. Of the 10 selected families with EM mothers, half were sired by EM fathers and half by LM fathers (giving 5 EM_M_EM_P_ and 5 EM_M_LM_P_ families). Similarly, of the 10 families with LM mothers, 4 were sired by EM fathers and 6 by LM fathers (giving 4 LM_M_EM_P_ and 6 LM_M_LM_P_ families). As for the mothers, there was no difference in body size and body condition (reproductive investment was not measured for males) between the EM and LM categories of father chosen for the experiment (*t*-tests for both comparisons, *P* > 0.6, Table 1).

### Offspring rearing conditions

Eggs were monitored every 1–2 days and once eye-spots became visible, eggs from each of the 20 families were transferred to the University of Glasgow (5th March 2013). Each family was kept in a separate egg basket, and was monitored daily until they were ready to begin exogenous feeding as juveniles (i.e., after complete metabolization of the maternal yolk sac, hereafter referred to as the “first-feeding stage”). This date of first feeding (12th April 2013±2 days) did not differ with respect to maternal or paternal maturation age. Water temperature was increased slowly throughout the period up to first feeding to reflect the seasonal changes that would occur in the wild, so that it reached ~13 °C (initial temperature ~6 °C, overall mean = 9.3 °C ± 0.2 SE) by the time the juveniles reached the first feeding stage.

On reaching the first feeding stage, groups of 50 sibling juveniles from each family were transferred to circular 5-L plastic containers (sides and floor replaced with stainless steel mesh) that were suspended in 1 of 2 re-circulating 1 m^2^ tanks. The position of the holding containers within each tank was changed randomly every 4–5 days to reduce the possibility of “within-tank” effects. Approximately 20% of the water in each tank was changed every 2–3 days during routine cleaning, and juveniles were fed ad libitum amounts of chopped bloodworm and powdered food daily (Micro Harmony, EWOS, West Lothian, Scotland). The top of each tank was covered with semi-transparent black material to provide overhead shelter. The juveniles were held in these conditions until they were measured for standard metabolic rate (SMR) and assayed for behavior (average water temperature, 12.7 °C ± 0.1 SE). One EM_M_EM_P_ family incurred high mortality in the rearing tanks (30% by the conclusion of the experiment) and so was excluded from the remainder of the experiment. Excluding this family, stock mortality from the first-feeding stage until the conclusion of the experiment (when juveniles were 5 weeks old) was low and similar among offspring from parents who matured at different ages (mean percent mortality ± SE, EM_M_ groups 9.3% ± 1.2, *n* = 9 families; LM_M_ groups 8.0% ± 1.0, *n* = 10 families; EM_P_ groups 7.8% ± 1.2, *n* = 8 families; LM_P_ groups 9.3% ± 1.0, *n* = 11 families, general linear model using arc-sin transformed mortality data; maternal maturation age, *F*_1,15_ = 0.68, *P* = 0.42; paternal maturation age, *F*_1,15_ = 1.06, *P* = 0.32; maternal × paternal maturation age, *F*_1,15_ = 0.002, *P* = 0.97).

### Measurement of offspring phenotypes

#### Respirometry

SMRs were measured in juveniles 16–33 days after they reached the first feeding stage (by which age they were robust enough to withstand the handling and short-term food deprivation required for measurement of metabolism), using flow-through respirometry. The oxygen consumption rates of 18 juveniles were measured on each day of testing; the fish were selected such that an even number of EM_M_ and LM_M_ juveniles from different families were screened each day (it was not possible to simultaneously screen an equal number of offspring from the 2 paternal maturation ages, so maternal maturation age was prioritized since maternal effects on offspring were presumed to be more likely than paternal). Prior to respirometry the fry were anaesthetized and marked with different color Visible Implant Elastomer tags (VIE, Northwest Marine Technology, Washington). Each fish was given a single mark between the dorsal fin and lateral line and the color code was alternated so that subsequent behavioral observations (see below) were conducted blind with respect to parental phenotype. A minimum of 4h after elastomer marking, the juveniles were placed in 20mL polypropylene respirometry chambers through which O_2_-saturated and UV-sterilized water was pumped at a constant rate by a peristaltic pump from a header tank. The fry were left to acclimate in the chambers overnight and measurements commenced 16–20h later, by which time they had settled and evacuated their guts. Previous studies of juvenile salmon have demonstrated a stable oxygen consumption rate after this period of acclimation (Metcalfe et al. 1995). To minimize activity, juveniles were kept in semi- darkness by placing a black cloth over the respirometry chambers, while low flow rates (average 0.16 l/h ± 0.0001 SE) removed the need for active swimming. Flow rates were calculated by collecting the water outflow from each chamber in a beaker over a measured time period (minimum 2min) and then weighing it (to 0.001g). Water temperature during respirometry averaged 12.4 °C ± 0.03 SE The reduction in water oxygen concentration due to juvenile respiration was measured with a Fibox 3 temperature-compensated oxygen meter (Loligo Systems, Tjele, Denmark). A flow-through fiber-optic cell with integrated planar oxygen sensor (PSt3 oxygen sensitive coating, Presens, Regensburg, Germany) was connected temporarily to the outflow of each respirometry chamber. The flow-through cell was calibrated with a 2-point calibration of oxygen-free water and oxygen-saturated water. Oxygen-free water was prepared by dissolving ca. 1g sodium sulphite (Na_2_SO_3_) in ca. 100mL of water. Oxygen-saturated water was prepared by simultaneously stirring and aerating ca. 100mL of header tank water. Metabolic rates (*V*O_2_, mL O_2_/h) of individual fish were calculated according to the equation:

$$V{Ο}_{2}=V_{W}\cdot \Delta C_{W}\cdot \beta {Ο}_{2}$$

where *V*_*W*_ is the flow rate (L/h) of water through the respirometry chamber, Δ*C*_*W*_ is the percentage difference in oxygen concentration between water in-flow and out-flow and βO_2_ is the capacitance of oxygen in the water. The oxygen concentration of water flowing into each chamber was determined in reference to the water exiting an empty (fish-less) control chamber. Measurements of the control chamber were made at the beginning and conclusion of each day. Two measurements of the control chamber were made to confirm that the O_2_ saturation of the inflow water, and the performance of the O_2_ sensor, were stable throughout the day. Measurements of outflow water oxygen concentration and temperature were logged (software Oxyview PST3v602, Presens, Regensburg, Germany) every 10s for each fish over a 5min period (minimum) or until the oxygen concentration had stabilized (to account for fluctuations caused by switching the sensor from 1 chamber to the next). Two to 3 replicate measurements of metabolic rate were made for each juvenile between 08:30 and 15:30h (with a minimum interval of 90min between measurements). The replicate measurements of SMR (when expressed as total oxygen consumption per individual) were highly repeatable within individuals (intraclass correlation coefficient = 0.69, 95% confidence interval: 0.62–0.75, calculated from variance components of a one-way Anova variance using the ICC package [Wolak et al. 2012] in the R Computing Environment [R Core Team, 2013]). Individual SMR was calculated as the average of these measurements and so includes possible diurnal variation in energy use. The juveniles were anaesthetized and weighed (to 0.001g) after measurements of metabolic rate.

#### Measurements of juvenile behavior

After being screened for metabolic rate, the juveniles in each day’s batch were allocated into pairs containing 1 individual from an EM mother and 1 from an LM mother (again prioritizing the importance of maternal age at maturation over paternal). Each replicate pair was chosen so that offspring from a given EM_M_ family were partnered with offspring from a different LM_M_ family in each batch of 18 fish, with each fish being used only once. Each pair was then placed in a compartment within a stream tank that functioned as a simulated feeding territory. Each compartment measured 20×12.5cm (water depth 14cm) and had sides of opaque Foamex (far wall) and glass (near wall, to allow observations) and upstream and downstream walls of mesh to allow water flow. The juveniles were then allowed to acclimate for 2 days prior to a 2-day period of behavioral observations. During the acclimation period, the juveniles were regularly fed chopped pieces of bloodworm that were pippetted beneath the water surface at the upstream end of each compartment. Over the 2-day period of observations we recorded the ability of the 2 fish to compete for items of food, together with the incidence of aggressive interactions. These measurements of behavior are referred to as competitive ability and aggression hereafter. The area of these simulated feeding territories (0.025 m^2^) closely approximated the estimated territory size (0.026 m^2^) required by juveniles of the size used in the current experiment, ca. 30mm fork length (Grant and Kramer 1990), thereby increasing the likelihood that the fish would compete. Water re-circulated through the tank at a water velocity of 1.3cm/s (estimated by tracking floating particles of foam). The feeding territories had a smooth floor to aid removal of uneaten food and feces. Behavioral trials were conducted in a temperature-controlled room (average water temperature 12.9 °C ± 0.02 SE).

The competitive ability of juveniles in each pair was measured 6 times daily by introducing a single piece of bloodworm (ca. 1–2mm long to prevent satiation of appetite) to each pair using the same technique employed during the acclimation period. The competitive ability of a given individual was thus calculated as the total number of food items acquired over the two day observational period. This produced 12 contest records for each juvenile, with the total possible count ranging from 0 to 12. During, and for a period of 1min after the measurement of competitive ability, we also recorded the initiators of any overt aggressive interactions (chasing and/or biting) in each pair. Aggression was thus calculated as the total number of aggressive interactions initiated by a given individual over the 2 day observational period. These observations revealed that overt displays of aggression were observed in more than 91 % of pairs and a hierarchy formed quickly between the 2 fish. Out of the total of 190 fish screened for metabolic rate and behavior, 6 died of unknown causes during the experiment (2 EM_M_, 4 LM_M_). Data from these individuals (and their partners) were excluded from the analysis. Behavioral and metabolic rate data were obtained for 7 to 12 juveniles from each of the 19 families retained in the experiment (*n* = 178 total individuals, *n* = 89 pairs, data collected over the first 5 weeks of life). All procedures were carried out under the approval of the UK Home Office (Project licence 60/4292).

### Data analysis

#### Offspring size

The size of juveniles from the EM and LM parents used in this experiment was assessed by comparing the initial size of offspring (i.e., egg size, which is the strongest predictor of hatchling body size in fish [Chambers and Leggett 1996]) and the size of the juveniles that were measured for metabolic rate (i.e., 16–33 days after consuming their endogenous reserves of yolk). Variation in egg size (log transformed) was modeled using linear mixed models with respect to the maternal maturation age of their mothers only (i.e., EM_M_ or LM_M_), because eggs were fertilized *after* measurements of size were made. However, the analysis of juvenile size (log-transformed) also included paternal maturation age (plus the interaction with maternal maturation age), juvenile age (days elapsed since exhaustion of the maternally provided yolk-sac, log transformed), and mean family egg size (also log-transformed) as additional explanatory variables, meaning that among-juvenile differences in age and initial size were taken into account. In both models, “family” was included as a random intercept to account for the nonindependence of measurements made on siblings.

#### Offspring metabolic rate and behavior

Variation in SMR (mL O_2_/h, transformed to log-scale) of the juveniles was modeled in response to the maturation age of each of their parents (i.e., EM or LM, as well as the interaction between maternal and paternal maturation age) using a linear mixed model, with body mass (also log-transformed) and measurement temperature as explanatory variables. “Family” and “measurement run” were fitted as random effect terms.

The aggression and competitive ability of offspring from EM and LM mothers were analyzed using models including the age of the individuals comprising each pair (days since the first feeding stage of development), plus the difference in body mass and difference in residual SMR (i.e., the residuals from a multiple regression of SMR [mL O_2_/h], on body mass [both transformed to log-scale] and measurement temperature, referred to hereafter as rSMR) between the EM_M_ individual and LM_M_ individual in each pair being fitted as explanatory variables. Preliminary exploration of the aggression and competitive ability data revealed that the counts of aggression approximated a Poisson distribution, whereas the competitive ability data (i.e., counts of food items consumed) were more normally distributed. The aggression data were thus modeled with a Poisson error structure (i.e., as a generalized linear mixed model), while a linear mixed model was used for the competitive ability data. In the generalized linear mixed model describing variation in offspring aggression, overdispersion was accounted for by fitting an additional observation-level random effect term to the full model (Zuur et al. 2012). In these analyses of juvenile behavior, we standardized the continuous explanatory variables (to aid model convergence) before fitting a full model, including the two-way interaction term between the difference in residual SMR and the difference in body mass between the EM_M_ and LM_M_ juveniles in each pair. Again “Family” was fitted as a random effect term to account for the nonindependence of measurements made on siblings. Lastly, we investigated the possible contribution of paternal maturation age to offspring behavior by repeating the behavioral analyses with paternal maturation age as an explanatory variable in place of maternal maturation age. Differences in behavior between juveniles from EM and LM fathers were analyzed with a smaller data set (*n* = 42 pairs) than the main analysis, because the pairs of juveniles had been established with a focus on measuring differences among pairs of offspring whose mothers differed in their age of maturation. Thus by chance, both juveniles in some pairs came from fathers of the same maturation age. Data from such pairs was therefore excluded from the analysis.

In all analyses, we used likelihood ratio tests (LRTs) to sequentially compare the log-likelihoods of simpler, nested models (using maximum likelihood). Terms were excluded at each stepwise iteration if the increase in the log-likelihood ratio statistic was not statistically significant (*P* > 0.05). Final models were re-fitted with restricted maximum likelihood. All statistical models were validated to check that underlying assumptions were satisfied; normality of residuals was assessed by plotting standardized residuals against the fitted values and explanatory variables from each model. All statistical analyses were conducted using the lme4 package (Bates et al. 2014) in R (R Core Team 2013).

## RESULTS

The eggs produced by LM mothers were slightly larger than those produced by EM mothers. However, this difference was not statistically significant (parameter estimate for log-transformed size of LM_M_ eggs compared to EM_M_ eggs ± SE, 0.03±0.02, *t*-value = 2.07, *P* = 0.054, EM_M_ egg size: mean 90.68, range 75.7–107.0, SD 6.88mg; LM_M_ egg size: mean 97.71, range 80.9–117.2, SD 8.22mg). By the time they were measured for rSMR and behavior, that is after 16–33 days of receiving exogenous food in our hatchery, EM_M_ and LM_M_ juveniles remained of similar size (linear mixed model with family as a random effect, maternal maturation age LRT, df = 1, χ^2^ = 2.09, *P* = 0.15), even when controlling for differences in initial (i.e., egg) size and juvenile age (parameter estimate ± SE log-egg size, 0.76±0.26, *t*-value = 2.95, *P* < 0.01, log-juvenile age, 0.66±0.05, *t*-value = 14.24, *P* < 0.0001, EM_M_ juvenile size: mean 185.6, range 109–284, SD 39.1mg, LM_M_ juveniles size: mean 188.3, range 121–321, SD 41.4mg). Juvenile size was not related to paternal maturation age (LRT, df = 1, χ^2^ = 0.01, *P* = 0.92) nor the interaction between paternal and maternal maturation age (LRT, df = 1, χ^2^ = 0.40, *P* = 0.53).

Juvenile metabolic rate was positively related to body mass and the average water temperature during measurement of metabolic rate (linear mixed model with family and measurement run as random effects, parameter estimates ± SE and corresponding *t*-values for each variable: log-body mass, 0.91±0.07, *t*-value = 13.27, *P* < 0.0001; temperature, 0.05±0.02 °C, *t*-value = 2.51, *P* = 0.013). No relationship between paternal or maternal maturation ages (or their interaction) and offspring SMR was evident (maternal × paternal maturation age LRT; df = 1, χ^2^ = 0.62, *P* = 0.43, maternal maturation age LRT; df = 1, χ^2^ = 0.67, *P* = 0.41, paternal maturation age LRT; df = 1, χ^2^ = 0.02, *P* = 0.90).

**Figure 1:**
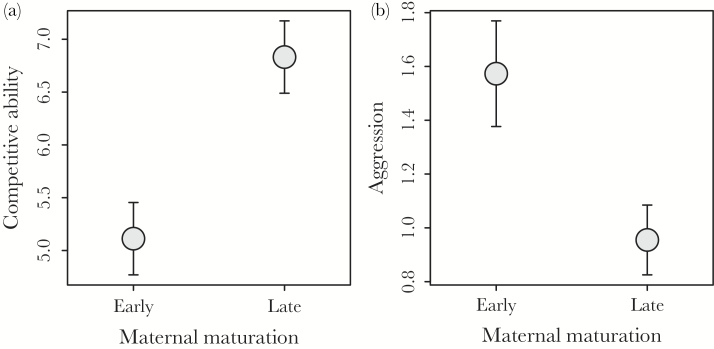
Mean differences (±SE) in (a) competitive ability and (b) aggression among offspring of female salmon that had matured early (EM_M_) or late (LM_M_). Data from pairs consisting of 1 juvenile from an EM mother and 1 juvenile from an LM mother competing for food and a feeding territory in sections of a stream tank. Competitive ability was calculated as the number of food items acquired by an individual over the 2 days of observation. Aggression refers to the total number of aggressive acts perpetrated by an individual during the observation period. See text for full details.

When comparing offspring behavioral traits, all explanatory variables except for maternal maturation age were nonsignificant and so removed from the analyses describing variation in offspring aggression and competitive ability (see Table 2 for summary of terms excluded from the 2 analyses). Thus, juveniles from LM mothers were on average better at competing for food than juveniles from EM mothers (linear mixed model with family as a random effect, parameter estimate ± SE and corresponding *t*-value for LM_M_ offspring compared to EM_M_ offspring, 1.75±0.64, *t* = 2.74, *P* = 0.014, see Figure 1a). However, when comparing the outcome of aggressive interactions within pairs we found that this asymmetry in behavior was reversed: juveniles from EM mothers were more aggressive than their LM counterparts (Poisson generalized linear mixed model with family as a random effect, parameter estimate ± SE and corresponding *z*-value for LM_M_ offspring compared to EM_M_ offspring, −0.49±0.20, *z* = −2.39, *P* = 0.017, see Figure 1b). Paternal age at maturation did not explain a significant amount of variation in the aggression and competitive ability of offspring (see Table 3).

**Table 2:** Summary of explanatory variables and LRTs used to exclude them from the mixed effect model analyses of juvenile aggression and competitive ability

Explanatory variable	χ^2^	df	*P*-value
Juvenile aggression			
Relative SMR × relative body mass	0.05	1	0.82
Relative SMR	0.05	1	0.82
Relative body mass	0.93	1	0.33
Juvenile age	1.30	1	0.25
Juvenile competitive ability			
Relative SMR × relative body mass	0.02	1	0.90
Relative SMR	0.02	1	0.90
Relative body mass	0.24	1	0.63
Juvenile age	0.004	1	0.95

Maternal maturation age was the only term retained in each model. See text for further details

**Table 3:** Summary of statistical models comparing the competitive ability and aggression of juvenile salmon sired by either EM or LM fathers

Response variable	Parameter estimate ± SE	*t*- or *z*-value	*P*-value
Competitive ability	0.54±0.99	0.55	0.59
Aggression	−0.31±0.38	−0.81	0.42

Competitive ability data were analyzed with a Gaussian linear mixed model, whereas the aggression data were analyzed with a Poisson generalized linear mixed model. Parameter estimates are given as treatment contrasts with respect to juveniles originating from EM fathers. Thus a positive value, e.g., for competitive ability, indicates that LM sired offspring would be more competitive than EM sired offspring. Note that neither of the comparisons presented are statistically significant.

**Figure 2:**
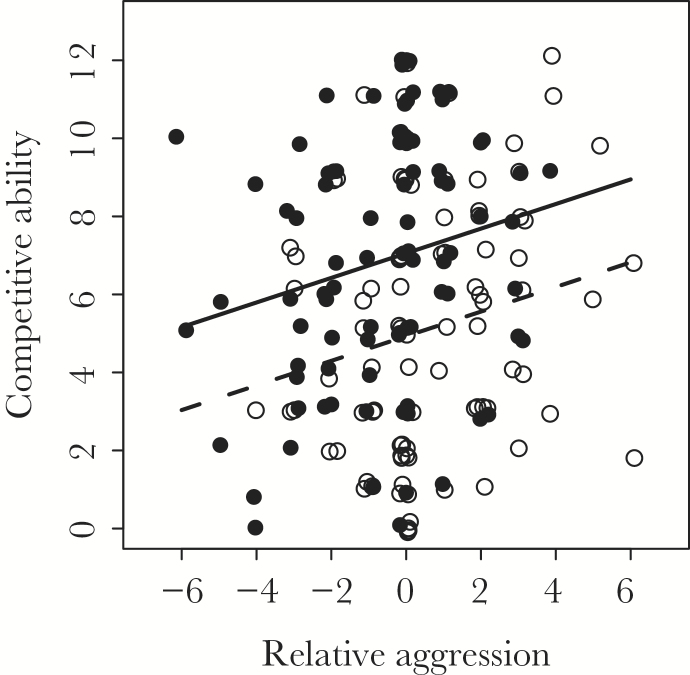
Relationship between competitive ability and relative aggression in juvenile offspring of EM (open dots, dashed line) and LM (filled dots, solid line) mothers. Relative aggression refers to the difference in aggression score between an individual and its partner. Lines represent predicted values from a linear mixed model; data points have been jittered to aid interpretation. See text for full details.

To better understand these contrasting asymmetries in behavior, we performed a follow-up analysis to investigate how juveniles from LM mothers were able to be better competitors (i.e., able to get a bigger share of a limiting food resource) despite being less aggressive on average than juveniles from EM mothers. Using a linear mixed model (with family set as a random effect term) we modeled variation in the competitive ability of individuals (calculated in the same way as the main analysis), with each individual’s age (days since the first feeding stage of development), the maturation age of their mothers (i.e., EM_M_ or LM_M_) and the difference in aggression between each individual relative to its partner, being fitted as explanatory variables. Overall, there was a positive relationship between competitive ability (i.e., the number of successful feeding attempts) and the difference in aggression between each of the individuals in a pair: the more aggressive member of a pair tended to obtain more food (parameter estimate ± SE, 0.32±0.12, *t*-value = 2.72, *P* < 0.01, see Figure 2). However, for a given level of relative aggression, juveniles from LM mothers obtained more food than did juveniles from EM mothers (parameter estimate ± SE for LM_M_ offspring compared to EM_M_ offspring, 2.13±0.59, *t*-value = 3.59, *P* < 0.01, see Figure 2).

## DISCUSSION

Here we present evidence that, while paternal maturation age had no noticeable effect, maternal maturation age was linked to distinct behavioral variation in offspring. This effect was observed despite the absence of differences in juvenile body size or timing of first-feeding between the 2 maternal maturation age groups (note that all eggs were fertilized within a 3-day period, and there were no differences between maternal types in the timing with which their offspring reached the first-feeding stage of development). In partial support of our hypothesis, juvenile offspring from mothers that matured at a younger age were more aggressive. Unexpectedly, though, they were less successful in competing for food than the offspring from mothers that matured later in life. Moreover, this dichotomy persisted even when controlling for the difference in aggression between the EM_M_ and LM_M_ offspring in each pair of fish. We also hypothesized that variation in SMR might underpin the growth differences among EM_M_ and LM_M_ juveniles previously reported from an experiment in natural conditions (Burton et al. 2013). We did not, however, observe a higher SMR in EM_M_ juveniles as predicted, suggesting that other factors might underlie the superior growth performance previously reported for EM_M_ juveniles relative to LM_M_ juveniles (Burton et al. 2013). For example, they might differ in the efficiency of digestion and growth or in the hormonal regulation of growth: some individuals are able to consume more food or process meals at a faster rate than others, meaning that they might be able to better capitalize when resources are abundant (Millidine et al. 2009; Auer et al. 2015). Individuals can also differ markedly in the efficiency with which they convert an ingested ration of food into new tissue (McCarthy et al. 1994), and relatively small juveniles have also been shown to “close the gap” in body size between themselves and larger conspecifics by upregulating expression levels of the growth hormone receptor (*GHR*) gene (Segers et al. 2012).

In salmonid fishes, life history variation in females is strongly linked with differences in the size of the offspring produced, presumably because of differences in the quantity or quality of resources that the female is able provide for each egg (Thorpe et al. 1984; Jonsson et al. 1996). Our results suggest that this influence may extend to behavioral traits in those offspring. Why? In salmon, it is possible that EM and LM females produce offspring that express different behaviors that are suited to different ecological niches. It has recently been shown that patterns of territorial defence in the closely related brown trout *Salmo trutta* depend on both the migratory history of the parents and the early environment experienced by the offspring, with offspring of migratory parents being more aggressive in defence of territories than offspring of residents, but only when the offspring have been reared at intermediate levels of food availability (Van Leeuwen et al. 2015). In the present case, high altitude tributaries are likely to have lower fish densities (Bohlin et al. 2001) and are known to produce a higher proportion of older smolts and thus LM adults (Shearer 1992), presumably because poorer growth conditions increase the time it takes for an individual to reach the size threshold required for seaward migration (Metcalfe and Thorpe 1990; Baum et al. 2004). Hence, LM females may be more likely to come from colder/more oligotrophic streams, where the combination of a lower fish density and poorer food supply might mean that the ability to obtain what food is present (referred to here as competitive ability) might have more direct impact on an individual’s growth performance than its tendency to direct aggression towards conspecifics. Conversely, EM females may be more likely to originate from more productive/eutrophic tributaries where conspecific densities are higher and hence aggression may be more important in securing a feeding territory and thus long-term access to food. Alternatively, if EM and LM parents originate from the same natal habitat, the behavioral differences among EM_M_ and LM_M_ juveniles presented here may reflect a within-tributary “counter-balancing strategy” (sensu Thorpe et al. 1984). In natural populations, there is a general lack of understanding as to how parental influences (whether they be genetic or environmental) on offspring size and behavior might interact. For example, we have previously documented a counter-intuitive pattern in the growth of juveniles in our study population: EM_M_ juveniles, found here to be more aggressive, were observed to grow faster under natural conditions than LM_M_ juveniles that were initially larger (Burton et al. 2013). Indeed, if less aggressive individuals are more likely to flee (an option not available in the conditions of the current study) than resist, high aggression could be advantageous for small juveniles (Svensson et al. 2012). Nevertheless, we caution against generalizing upon the adaptive nature of behavioral variation measured in laboratory conditions (Niemelä and Dingemanse 2014) since there is growing evidence that links between behavior and life history traits might be less consistent than previously assumed and can vary among life stages or environments (Adriaenssens and Johnsson 2013). When considering the mechanistic basis of our results, we would first like to emphasise that we cannot be certain that the reported patterns in offspring behavior are driven by maturation age per se or another correlated variable. Nevertheless, environmental conditions experienced by juvenile salmon should be very similar across generations because adults generally home with great accuracy to spawn in their natal stream and do so within a narrow seasonal window (Fleming 1996).

While substantial genetic differentiation has been reported among Atlantic salmon sampled from different tributaries within the same river system (Primmer et al. 2006), our results are unlikely to reflect local adaptation since the parental fish came from eggs that had been randomly mixed and then distributed by hand among tributary streams of the Conon catchment (there being no natural spawning due to the presence of hydropower dams). However, this means that our results could be attributed to trans-generational plasticity given the relatively high degree of “predictability” in the juvenile environment from year to year (a prerequisite for trans-generational plasticity to be adaptive, Burton and Metcalfe 2014). Indeed, evidence from several vertebrates demonstrates that behavioral phenotypes can be transmitted directly from one generation to the next (Doumas et al. 1994; Francis et al. 1999; Müller et al. 2011), with epigenetic regulation of specific genes (Weaver et al. 2004; McGowan et al. 2009) or alteration of egg components (McCormick 1998; Eising et al. 2006) representing possible mechanistic pathways. The possible contribution of trans-generational plasticity to such phenomena could be investigated experimentally by behavioral phenotyping of juveniles derived from parental stock (sourced from a single population and kept in common conditions for several generations) that have been subject to experimental manipulation of maturation age, for example by dietary or temperature alteration. This approach could also be designed to assess possible mechanistic pathways, for example, by measuring *GHR* expression in juveniles and quantifying levels of egg hormones or antioxidants. Such data will be key to better understanding the contribution of local adaptation and the environment to behavioral variation arising from parental maturation age and the ecological consequences of maturation age in general.

## FUNDING

This work was supported by the Natural Environment Research Council (grant number: NE/I025182/1) with additional support to N.B.M. from the European Research Council (Advanced Grant 322784).
